# Thromboembolic events associated with antiangiogenic monoclonal antibodies: a disproportionality analysis from FDA adverse event reporting system (FAERS) database

**DOI:** 10.1186/s12959-026-00829-w

**Published:** 2026-01-17

**Authors:** Hong Zhou, Yanzhao Su, Jianrong Song, Lan Zhen

**Affiliations:** 1 Department of Pharmacy, Fujian Maternity and Child Health Hospital College of Clinical Medicine for Obstetrics & Gynecology and Pediatrics, Fujian Medical University, Fuzhou, Fujian China; 2 Department of Gynecology Oncology, Fujian Maternity and Child Health Hospital College of Clinical Medicine for Obstetrics & Gynecology and Pediatrics, Fujian Medical University, Fuzhou, Fujian China

## Abstract

**Background:**

Antiangiogenic monoclonal antibodies are increasingly used for various cancers. Although studies have reported thromboembolic events (TEEs) in patients receiving antiangiogenic monoclonal antibodies (mAbs), a comprehensive analysis of various thromboembolic events and their correlation with clinical outcomes is lacking.

**Objectives:**

To evaluate potential signals and clinical outcomes of thromboembolic events associated with antiangiogenic monoclonal antibodies.

**Design:**

An observational, retrospective, and pharmacovigilance study based on the FAERS database collected from the first quarter of 2004 to the third quarter of 2024 was conducted.

**Methods:**

The reporting odds ratio (ROR) and the Bayesian Information Criterion (IC) with 95% confidence intervals (CIs) were employed to assess the disproportionate reporting of TEEs linked with antiangiogenic mAbs. A multivariable logistic regression model was implemented to evaluate the association between TEEs and mortality outcomes.

**Results:**

A total of 3319 TEEs with antiangiogenic mAbs were identified including bevacizumab intravenously (3117 reports), ramucirumab (95 reports), and aflibercept intravenously (107 reports) from health professionals. All three antiangiogenic mAbs detected positive signals, including VTE, PE, ATE, and overall TEEs. Interestingly, no positive signals for myocardial Infarction were detected for all the three antiangiogenic mAbs, and no cerebral ATE was detected for aflibercept. Additionally, no signal was detected when comparing the antiangiogenic mAbs directly with one another. Multivariable logistic regression analysis revealed that fatal TEEs occurred more frequently compared to non-TEE outcomes. This association reached statistical significance in the subgroup of reports for bevacizumab in colorectal cancer.

**Conclusion:**

This post-marketing data revealed that antiangiogenic mAbs had similar risks of TEEs. However, the occurrence of TEEs was associated with a higher risk of mortality. Due to the inherent limitations of pharmacovigilance data, these findings represent signals that require validation in prospective studies to establish causality.

**Supplementary Information:**

The online version contains supplementary material available at 10.1186/s12959-026-00829-w.

## Introduction

As advancements continue to shape the landscape of cancer therapy, antiangiogenic therapy has emerged as a cornerstone in the management of various malignancies. Bevacizumab, ramucirumab, and aflibercept, as antiangiogenic monoclonal antibodies(mAbs), target the vascular endothelial growth factor (VEGF) pathway, demonstrating efficacy by inhibiting tumor angiogenesis and are widely used in the treatment of various cancer [1–3]. However, besides their therapeutic benefits, these drugs may be associated with the risk of inducing thromboembolic events.

Previous studies in cancer patients showed inconsistent results regarding the correlation between the use of bevacizumab and the occurrence of thrombosis [4–8], and limit data revealed that thrombosis might not be related to prognosis [9, 10]. A pooled analysis of five randomized controlled trials (RCTs) by Scappatici F et al. found a significant increase in arterial thromboembolic events (ATEs) with bevacizumab-chemotherapy combinations, but not venous thromboembolism (VTE) [8]. A meta-analysis of safety data from six global RCTs on ramucirumab suggested that the risks of ATE, VTE, and high-grade bleeding events, including gastrointestinal (GI) bleeding, were not significantly increased [11], potentially distinguishing ramucirumab among antiangiogenic agents [4, 12]. However, these findings are in contrast with another meta-analysis spanning 11 global RCTs [13]. Moreover, a single-institution retrospective analysis of 468 cancer patients treated with chemotherapy incorporating bevacizumab (*n* = 286), ramucirumab (*n* = 179), or aflibercept (*n* = 3) revealed no significant difference in the likelihood of thromboembolic events across cancer types, though differences were noted between the different antiangiogenic agents [14]. Despite the known association of antiangiogenic treatments with heightened thrombosis risk, comprehensive comparative analyses detailing the thrombotic profiles of these specific monoclonal antibodies and their impact on patient outcomes in real-world are lacking.

The FDA Adverse Event Reporting System (FAERS) database serves as a comprehensive resource for monitoring post-marketing drug safety and for studying real-world outcomes related to drug use [15]. While previous research has leveraged FAERS to assess various drug safety profiles, detailed analyses focusing on the thromboembolic risks posed by antiangiogenic agents and their impact on prognosis in cancer patients are sparse. This study leverages FAERS to explore the characteristics and prognosis of thromboembolic events associated with antiangiogenic mAbs as reported in the FAERS database.

## Materials and methods

### Data sources and collection

This study utilized data retrieved from the American Standard Code for Information Interchange(ASCII) report files of the FDA adverse event reporting system(FAERS) database, spanning from the first quarter of 2004 (coinciding with the initial U.S. approval of bevacizumab) to the second quarter of 2024 [16]. The data were imported into MySQL 8.0 and processed using Navicat Premium 17 software. Duplicates were removed adhering to criteria recommended by the FDA [17]. Adverse events (AEs) for each case were systematically coded in accordance with the Medical Dictionary for Regulatory Activities (MedDRA) [18], and mapped to Preferred Terms (PTs). The time to onset of AEs was calculated by subtracting the start date (START_DT field in the THER file) from the onset date (EVENT_DT field in the DEMO file) after excluding reports of input errors (inaccurate inputs, missing data, and EVENT_DT earlier than START_DT).

Venous thromboembolic events (VTEs), arterial thromboembolic events (ATEs), and unspecified and mixed events were identified using the MedDRA selected Preferred Terms (PTs) from the Standardised MedDRA Queries (SMQs) for Embolic and thrombotic events (SMQ = 20000081). Pulmonary embolism (PE), myocardial infarction (MI), and cerebral ATE were identified according to a comprehensive literature review and relevant PTs (Supplement Table 1) [19, 20].

**Table 1:** Clinical characteristics of patients with thromboembolic events associated with antiangiogenic monoclonal antibodies

Characteristics		Bevacizumab	Ramucirumab	Aflibercept
Total number of reported AEs		3117	95	107
Sex				
Female		1359(43.6)	32(33.7)	34(31.8)
Male		1310(42)	54(56.8)	59(55.1)
Unknown		448(14.4)	9(9.5)	14(13.1)
Age, yrs*		*n* = 2369	*n* = 75	*n* = 91
< 18		74(2.4)	0(0)	1(0.9)
18 ≥ and < 65		1187(38.1)	30(31.6)	36(33.6)
≥ 65		1108(35.5)	45(47.4)	54(50.5)
Unknown		748(24)	20(21.1)	16(15)
Outcome				
Fatal		949(30.4)	30(31.6)	19(17.8)
Non-fatal		2168(69.6)	65(68.4)	88(82.2)
Reported Countries				
United States		930(29.8)	21(41)	16(15)
Japan		677(21.7)	41(21)	21(19.6)
Germany		209(6.7)	3(3)	14(13.1)
France		198(6.4)	2(3)	22(20.6)
Tumour indications				
Colorectal cancer		1085(34.8)	11(11.6)	83(77.6)
Non-small cell lung cancer		258(8.3)	10(10.5)	0
Gastroesophageal cancer		35(1.1)	42(44.2)	4(3.7)
Ovarian cancer in 8.3%		258(8.3)	0	0
Glioblastoma in 7.3%		227(7.3)	0	0
Other cancers		1115(35.7)	20(21)	4(3.7)
Unknown		139(4.5)	12(12.6)	16(15)
Comorbidity				
Hypertension		135(4.3)	1(1.1)	7(6.5)
Atrial fibrillation		7(0.2)	0	0
Treatment strategy				
Plus chemotherapy		1970(63.2)	54(56.8)	80(74.8)
Other combined therapy		165(5.29)	0	0
Monotherapy		982(31.5)	41(43.2)	27(25.2)
Time to AE, days*		*n* = 2060	*n* = 54	*n* = 84
0-30d		660(32)	31(57.4)	42(50)
31-60d		370(18)	8(14.8)	8(9.5)
61-90d		205(10)	7(13)	5(6)
91-180d		382(18.5)	4(7.4)	13(15.5)
181-365d		248(12)	2(3.7)	13(15.5)
> 365d		195(9.5)	2(3.7)	3(3.6)
Median (interquartile range)		60.5 (IQR 21–153)	26.5 (IQR 11–61)	31.5 (IQR 9.75–127.5)

Values are n, n/N (%), or n (%). *Number of patients for whom data were available. AE = adverse event

The study targeted antiangiogenic monoclonal antibodies (mAbs) including bevacizumab, ramucirumab, and aflibercept. Given that aflibercept and bevacizumab are administered both intraocularly and intravenously, only reports of intravenous administration were considered. Reports with an unspecified route of administration were excluded from the analysis. The dataset was further screened to include only reports submitted by healthcare professionals (including physicians, pharmacists, and other health professionals), where antiangiogenic mAbs were regarded as the ‘primary suspect’ (Supplement Fig. 1).

### Statistical analysis

In this study, descriptive statistics were employed to elucidate the clinical characteristics of thromboembolic events (TEEs) associated with antiangiogenic mAbs as reported in the FDA Adverse Event Reporting System (FAERS) database. The time to onset of TEEs was compared using non-parametric tests: the Mann-Whitney U test for dichotomous variables and the Kruskal-Wallis test for datasets with more than two subgroups. A significance level was set at *p* < 0.05.

Furthermore, disproportionality analysis was conducted utilizing two consolidated measures, provided there were at least three reports: the reporting odds ratio (ROR) and the Bayesian information component (IC), each with corresponding 95% confidence intervals (CIs) [15, 21]. Established thresholds for signaling disproportionate reporting were applied, specifically, a lower limit of the 95% CI exceeding 1 for ROR and greater than 0 for IC, respectively. Detailed methodologies of the disproportionality analysis are presented in Supplement Table 2.

**Table 2:** Signal strength of antiangiogenic monoclonal antibodies related TEEs compared with the full database

ADR	Bevacizumab(intravenous)		Ramucirumab		Aflibercept(intravenous)	
	ROR (95% CI)	IC (95% CI)	ROR (95% CI)	IC (95% CI)	ROR (95% CI)	IC (95% CI)
PE	2.37(2.17–2.59)	1.23(1.01–1.45)	2.49(1.52–4.07)	1.31(0.7–1.68)	2.32(1.34-4)	1.21(0.55–1.6)
MI	0.64(0.56–0.73)	-0.64(-0.89-0.38)	0.39(0.15–1.04)	-1.35(-2.02-0.3)	1.47(0.85–2.53)	0.55(-0.02-1.03)
Cerebral ATE	1.38(1.2–1.6)	0.47(0.2–0.73)	2.42(1.3–4.5)	1.27(0.52–1.68)	1.66(0.75–3.7)	0.73(-0.11-1.31)
VTE	2.53(2.39–2.68)	1.32(1.12–1.52)	2.27(1.6–3.22)	1.18(0.72–1.52)	2.2(1.5–3.21)	1.13(0.65–1.48)
ATE	1.33(1.25–1.43)	0.41(0.2–0.62)	1.73(1.23–2.42)	0.78(0.36–1.14)	2.22(1.61–3.06)	1.14(0.71–1.47)
Unspecified and mixed	1.77(1.67–1.87)	0.81(0.61-1.0)	1.57(1.13–2.2)	0.65(0.24–1.01)	2.55(1.92–3.38)	1.33(0.93–1.64)
Overall TEEs	1.75(1.69–1.81)	0.78(0.61–0.95)	1.74(1.42–2.14)	0.78(0.46–1.08)	2.25(1.85–2.73)	1.14 (0.82–1.42)

Abbreviations: PE, pulmonary embolism; MI, myocardial infarction; VTE, venous thromboembolism; ATE, arterial thromboembolism; TEEs, thromboembolic events

A multivariable logistic regression model was developed to explore the association between TEEs and mortality outcome. This model adjusted for confounders such as age, sex, and type of drug, and the magnitude of association was expressed as an adjusted odds ratio (aOR) with its respective 95% CI. A P-value of < 0.05 was interpreted as statistically significant. All statistical analyses were conducted using IBM SPSS Statistics software (version 21.0).

## Results

### Descriptive characteristics

From the first quarter of 2004 to the third quarter of 2024, our research identified adverse events associated with antiangiogenic mAbs, with health professionals attributing bevacizumab, ramucirumab, and aflibercept as the primary suspected drugs in 92,055 reports, 2,814 reports, and 2,455 reports, respectively. Among the antiangiogenic mAbs-related AEs, we identified a total of 3,319 thromboembolic events (TEEs), which included 3,117 reports involving intravenous bevacizumab, 95 reports with ramucirumab, and 107 reports concerning intravenous aflibercept (Supplement Fig. 1). The primary indications for bevacizumab therapy were colorectal cancer in 34.8% of the reports, non-small cell lung cancer in 8.3%, ovarian cancer in 8.3%, and glioblastoma in 7.3%. For ramucirumab therapy, the indications were gastroesophageal cancer in 44.2% of the reports, colorectal cancer in 11.6%, and non-small cell lung cancer in 10.5%. For aflibercept, the indications were colorectal cancer in 77.6% of the reports and gastric cancer in 3.7%. Regarding treatment strategy, the antiangiogenic agents in combination with chemotherapy, accounted for 63.2% of bevacizumab reports, 56.8% of ramucirumab reports, and 74.8% of aflibercept reports. Cardiovascular comorbidities, such as hypertension and atrial fibrillation, were infrequently reported. The predominant demographic for ramucirumab and aflibercept was males aged 65 years and older. Bevacizumab usage was distributed over a wider age range, with more women than men due to its broad use across various tumor types-including ovarian cancer. After excluding ovarian cancer cases, its gender distribution for bevacizumab (female, 35.3%; male, 42%) aligned with that of ramucirumab and aflibercept. Bevacizumab and ramucirumab were mainly reported in the United States, while aflibercept was mainly reported in France and Japan. The detailed clinical characteristics of these patients are presented in Table 1.

### Time to onset analysis

**Fig. 1:**
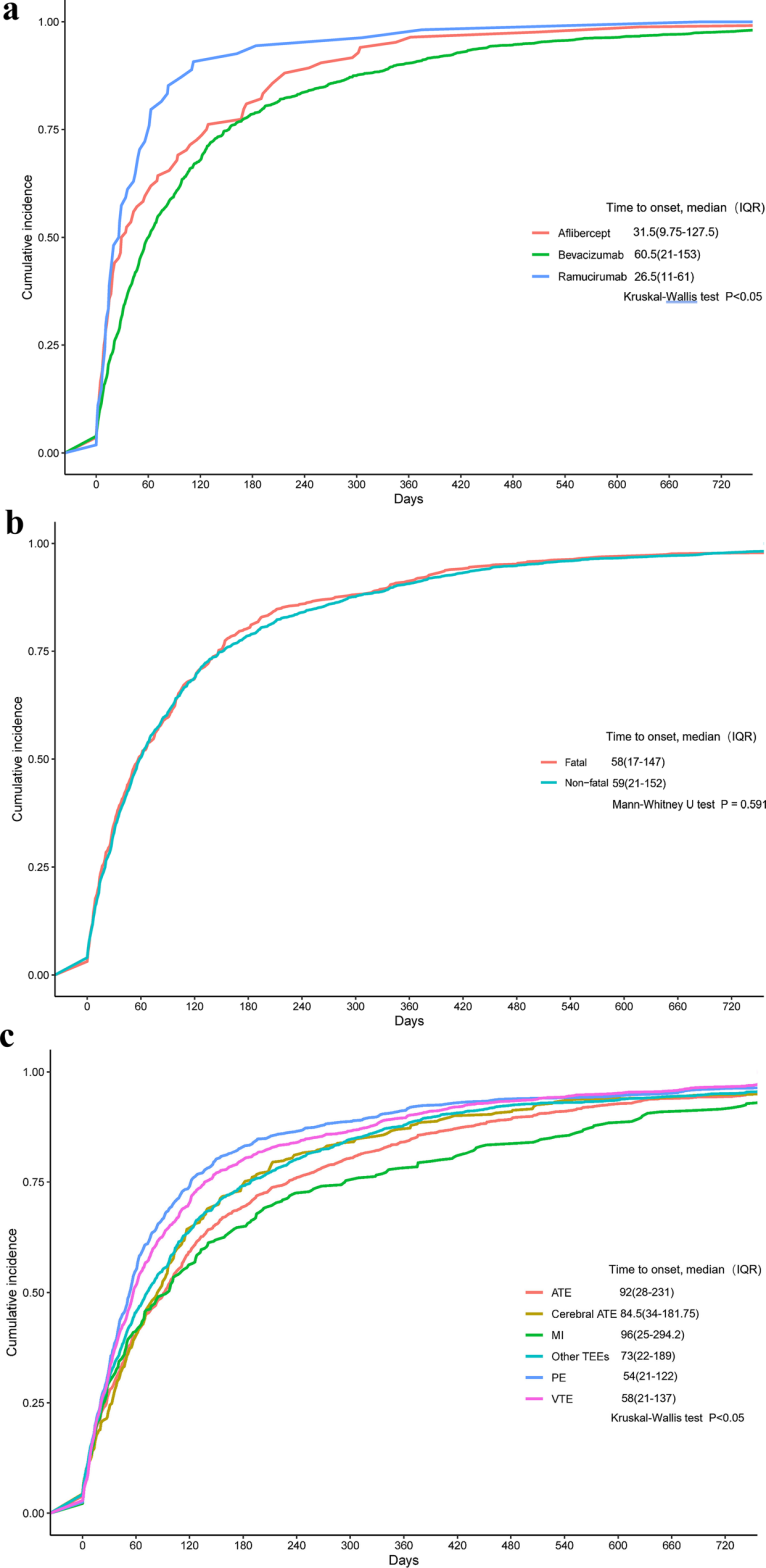
Cumulative incidence (%) of thromboembolic events since the initiation of antiangiogenic mAbs. Grouped by (**a**) different antiangiogenic mAbs, (**b**) different clinical outcomes, and (**c**) different types of TEEs

Compared to bevacizumab (60%) and aflibercept (65.5%), a higher proportion of TEEs associated with ramucirumab (85.2%) tended to occur within the first three months (Table 1). Patients receiving intravenous bevacizumab showed a median time to onset of 60.5 days (interquartile range, IQR, 21–153 days), compared to 26.5 days (IQR 11–61) for ramucirumab, and 31.5 days (IQR 9.75–127.5) for aflibercept. There was a statistically significant difference in the onset times among the groups, with bevacizumab having a longer time to onset than the other two agents, as analyzed by the Kruskal-Wallis test (*P* < 0.05) (Fig. 1a). Furthermore, the median time to onset for fatal TEEs was 58 days (IQR 17–147) and for non-fatal TEEs was 59 days (IQR 21–152), with no significant difference found using the Mann-Whitney U test (*P* = 0.591) (Fig. 1b).

Additionally, a comparison across different types of thromboembolic events also revealed a statistically significant difference, as analyzed by the Kruskal–Wallis test (*P* < 0.05). The median time to onset for ATE (including MI and cerebral ATE) was longer than that for VTE (including PE) (Fig. 1c).

### Disproportionality analysis

**Fig. 2:**
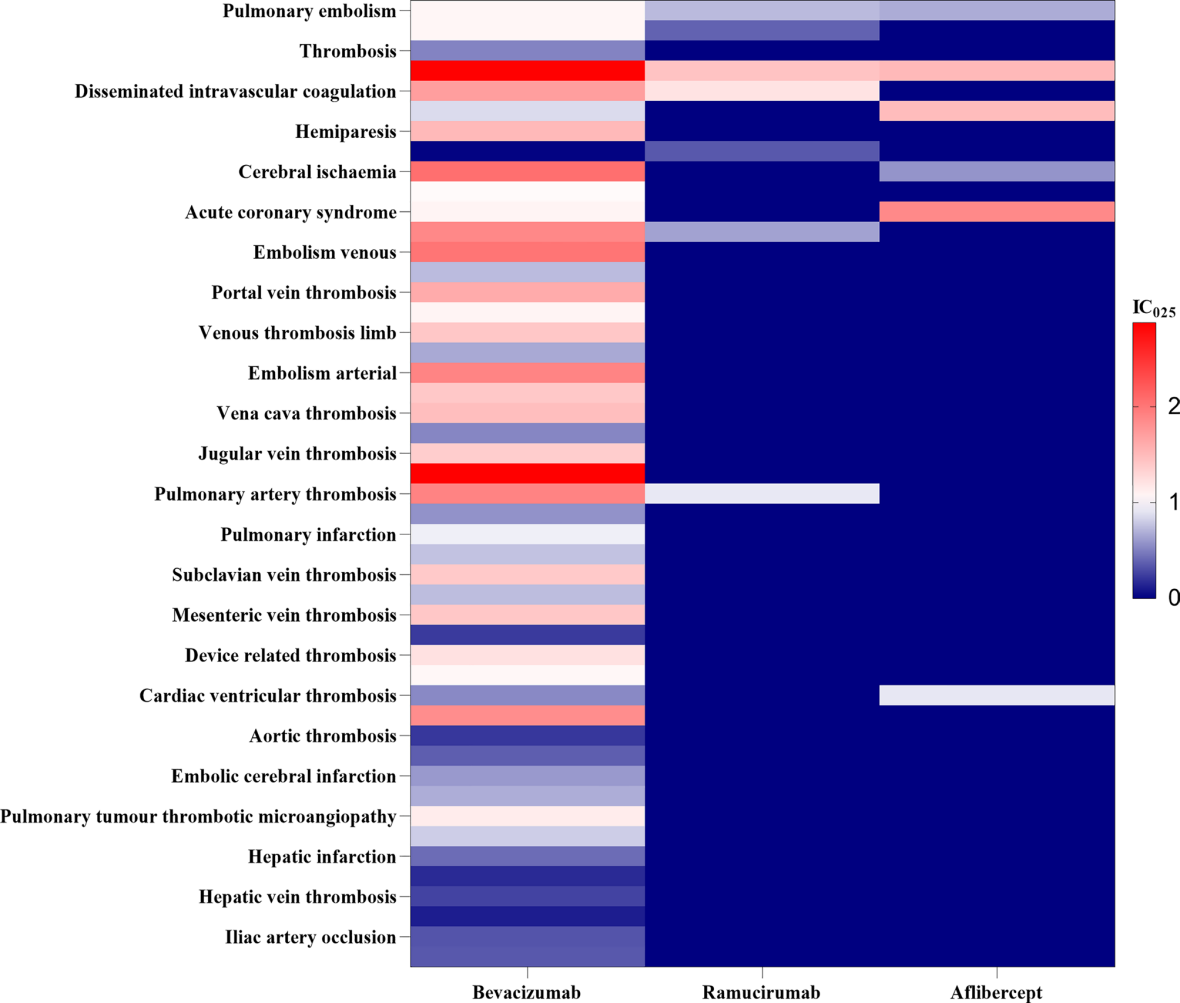
Thromboembolic events profile of different antiangiogenic mAbs drugs. Data are lower end of 95% confidence interval of IC compared with the full database. Statistically significant IC (i.e., IC025 > 0) are assigned with a red color

To spot the thrombotic events signals of antiangiogenic mAbs, we compared with the full database. The profiles of thromboembolic events associated with different antiangiogenic mAbs are detailed in Fig. 2. Bevacizumab was associated with 50 significant preferred terms (PTs), ramucirumab with 7 PTs, and aflibercept with 6 PTs.

The analysis identified significant associations with various thromboembolic events as shown in Table 2:

Bevacizumab, administered intravenously, displayed pharmacovigilance signals for VTE [ROR 2.53 (2.39–2.68); IC 1.32 (1.12–1.52)], PE [ROR 2.37 (2.17–2.59); IC 1.23 (1.01–1.45)], ATE [ROR 1.33 (1.25–1.43); IC 0.41 (0.20–0.62)], cerebral ATE [ROR 1.38 (1.20–1.60); IC 0.47 (0.20–0.73)], and overall TEE [ROR 1.75 (1.69–1.81); IC 0.78 (0.61–0.95)].

Ramucirumab showed signals for VTE [ROR 2.27 (1.60–3.22); IC 1.18 (0.72–1.52)], PE [ROR 2.49 (1.52–4.07); IC 1.31 (0.70–1.68)], ATE [ROR 1.73 (1.23–2.42); IC 0.78 (0.36–1.14)], cerebral ATE [ROR 2.42 (1.30–4.50); IC 1.27 (0.52–1.68)], and overall TEE [ROR 1.74 (1.42–2.14); IC 0.78 (0.46–1.08)].

Aflibercept, administered intravenously, exhibited positive signals for VTE [ROR 2.20 (1.50–3.21); IC 1.13 (0.65–1.48)], PE [ROR 2.32 (1.34-4.00); IC 1.21 (0.55–1.60)], ATE [ROR 2.22 (1.61–3.06); IC 1.14 (0.71–1.47)], and overall TEEs [ROR 2.25 (1.85–2.73); IC 1.14 (0.82–1.42)]. However, no signals were detected for cerebral ATE.

Importantly, no positive signals for MI were identified across the three monoclonal antibodies (mAbs). Additionally, when comparing the three antiangiogenic monoclonal antibodies directly with one another, no significant signals were detected (see Table S3).

### Mortality rates associated with TEEs

**Fig. 3:**
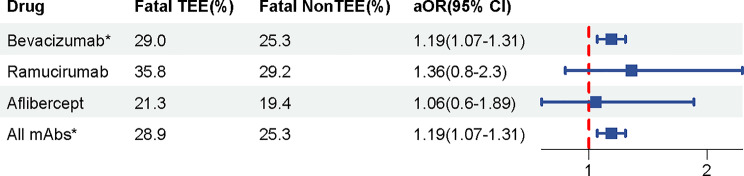
The association between TEEs and the death outcomes. Association was adjusted for age, sex, and drug type, and the effect size represent fatal TEEs versus non-TEE. Abbreviations: aOR, adjusted odds ratio; and CI, confidence interval. *P < 0.05

Pulmonary embolism was the most commonly reported fatal adverse event, accounting for 180 cases (18.04%). The association between TEEs and the death outcomes are showed in Fig. 3. Overall, mortality was significantly higher in cases involving TEEs compared to those without TEEs [28.9% vs. 25.3%, aOR 1.19 (1.07–1.31), *P* < 0.001], adjusted for sex, age and different drugs. This significant difference was observed in patients receiving intravenous bevacizumab, who had a higher mortality rate compared to non-TEE reports [29.0% vs. 25.3%, aOR 1.19 (1.07–1.31), *P* < 0.001]. We further conducted subgroup analyses of bevacizumab-associated TEEs across different tumors, adjusted for sex and age. Specifically, for colorectal cancer, there was a significantly increased risk of mortality [aOR 1.40 (1.18–1.66), *P* < 0.001]. However, for non-small cell lung cancer, the increased risk was not statistically significant [aOR 0.97 (0.63–1.50), *P* > 0.05]. Similar non-significant trends were observed in ovarian cancer [aOR 1.41 (0.98–2.03), *P* > 0.05] and glioblastoma [aOR 0.78 (0.52–1.18), *P* > 0.05].

## Discussion

Antiangiogenic monoclonal antibodies, including bevacizumab, ramucirumab, and aflibercept, have become widely integral to cancer treatment strategies due to their ability to inhibit tumor angiogenesis. However, they may increase thrombotic events, and the association between thromboembolic events and prognosis is of concern. Previous disproportionality analyses focusing on anti-VEGF drugs have either excluded cancer patients with intraocular administration [22, 23] or overall safety profile or other specific adverse events in cancer patients [24–27]. Our study is the first large-scale post-marketing pharmacovigilance analysis of various thromboembolic risks specifically associated with the antiangiogenic monoclonal antibodies from the FAERS database. In the study, we found that antiangiogenic mAbs were linked to an increased risk of overall TEEs, including VTE, PE, and ATE, but not MI. Interestingly, aflibercept did not show an increased risk for cerebral ATE. Additionally, our data observed that bevacizumab increased the risk of mortality in colorectal cancer.

In the present study, of the three antiangiogenic mAbs, bevacizumab was substantially more reported and identified more signals in this study than ramucirumab and aflibercept, probably due to its being the first monoclonal antibody marketed for antiangiogenic therapy [28] and its widespread use in various tumors [29–32]. There were positive signals for increased TEEs with antiangiogenic monoclonal antibodies (mAbs). A direct comparison of the three drugs showed a similar risk of TEEs. Kanbayashi Y et al. conducted a retrospective analysis of cancer patients, comparing thromboembolic events between bevacizumab (*n* = 286, TEEs = 23), ramucirumab (*n* = 179, TEEs = 3), and aflibercept (*n* = 3, TEEs = 0), indicating that the likelihood of thromboembolic events varies among antiangiogenic agents [14]; however, a relatively small number of patients was analyzed. Therefore, a prospective multicenter study is needed to confirm these results.

Notably, our study showed that bevacizumab-related TEEs increased the risk of mortality, with no statistical differences observed between the ramucirumab and aflibercept groups. Bevacizumab targets the vascular endothelial growth factor (VEGF) pathway and differs slightly in its mechanism of action from ramucirumab and aflibercept, which may lead to differences [33]. Subsequent subgroup analyses revealed a statistically significant increased risk of mortality associated with colorectal cancer. Kanbayashi Y et al. revealed that colorectal cancer was observed as an independent risk factor for TEEs [14]. Conversely, non-significant trends in non-small cell lung cancer, ovarian cancer, and glioblastoma. Previously limited data revealed that the occurrence of thromboembolic events was not associated with overall survival in ovarian cancer [9] and malignant gliomas [10]. This finding suggests that the association between bevacizumab-related thromboembolic events and the prognosis of colorectal cancer merits further investigation.

Aflibercept is an antiangiogenic mAb with a broader mode of action, acting as a soluble receptor that binds to human vascular endothelial growth factor A (VEGF-A), VEGF-B, and the placental growth factor [34], and is approved for second-line metastatic colorectal cancer [1, 35]. There are few studies on aflibercept and thrombotic risk [36, 37] In the package insert [1], ATEs associated with aflibercept, including transient ischemic attack, cerebrovascular accident, and angina pectoris, occurred more frequently, and ATE was reported in 2.6% of patients. Interestingly, our data indicate that aflibercept had a risk for overall arterial thromboembolism, but did not carry the same risk for cerebral ATE, which was different from other agents in its class. In a retrospective multicentric study including 243 patients, FOLFIRI plus bevacizumab or aflibercept were compared in terms of efficacy and safety in patients with RAS-mutant colon cancer, suggesting that the side effects were higher in the aflibercept arm than bevacizumab arm [38]. A meta-analysis shows that the combination of aflibercept plus FOLFIRI offers better survival efficacies; however; it is also associated with more high-grade adverse events, with 5% of them being grade 3–4 venous thromboembolic events [39]. Currently, there is limited data on the correlation between aflibercept and thrombosis, indicating a need for further research.

A recent analysis of the FAERS database by Tang et al. [40] investigated TEEs associated with vascular endothelial growth factor receptor tyrosine kinase inhibitors (VEGFR-TKIs), providing a critical parallel to our study of antiangiogenic mAbs. While Tang et al. identified MI as the most frequently reported and strongly signaled arterial TEE for several VEGFR-TKI, our analysis of antiangiogenic mAbs did not yield a significant disproportionality signal for MI. Antiangiogenic mAbs target the VEGFR, whereas multitargeted TKIs inhibit both the VEGFR and the platelet-derived growth factor receptor (PDGFR) [41]. This suggests a potentially distinct cardiovascular toxicity profile, possibly attributable to the more targeted extracellular ligand/receptor blockade of mAbs versus the broader intracellular kinase inhibition and off-target effects characteristic of multi-targeted TKIs [42].Our findings fill a gap in real-world evidence concerning TEEs associated with antiangiogenic drugs.

While our study provides significant insights into the association of thromboembolic events with antiangiogenic monoclonal antibodies, several limitations must be acknowledged. Firstly, the reliance on the FAERS database means our findings are subject to inherent biases common in spontaneous reporting systems, such as underreporting and overreporting. This could affect the accuracy of the reported incidence rates of thromboembolic events. Despite these constraints, our findings were derived from reports submitted exclusively by health professionals. Second, owing to the unavailability of exact causes of death and event time points, fatal outcomes must be interpreted as all-cause mortality rather than as direct consequences of the adverse events. Temporal uncertainties hinder causal inference, and scarce follow-up data limit insights into long-term prognosis. To enhance signal specificity for antiangiogenic mAbs, we focused on first identifying relevant AEs and then assessing their outcome associations. We utilized logistic regression to mitigate confounding bias, enhancing the reliability of the results. Moreover, the retrospective design of our disproportionality analysis inherently limits the ability to infer causality between the use of antiangiogenic mAbs and the outcomes of thromboembolic events. Given these constraints, the analysis is exploratory and precludes definitive conclusions; however, it delineates real-world prognostic patterns associated with adverse events. The safety signals identified as such merit rigorous evaluation in prospective studies.

In conclusion, our comprehensive pharmacovigilance analysis of the FAERS database identified positive signals between antiangiogenic monoclonal antibodies and thromboembolic events. However, aflibercept does not increase the risk of cerebral ATE. Additionally, different antiangiogenic mAbs had similar risks of TEEs. Notably, thromboembolic events associated with bevacizumab might affect the prognosis warranting further investigation. As antiangiogenic therapies become more prevalent in clinical settings, the potential for thrombotic complications necessitates vigilant monitoring and management to safeguard patient outcomes.

## Supplementary Information

Below is the link to the electronic supplementary material.

Supplementary Material 1

## Data Availability

The datasets generated and/or analyzed during the current study are available in the US FAERS database.
