# Supplementary material for: Thromboembolic events associated with antiangiogenic monoclonal antibodies: a disproportionality analysis from FDA adverse event reporting system (FAERS) database
Source: Thromb J. 2026 Jan 17;24:20. doi: 10.1186/s12959-026-00829-w (PMC12895867; doi:10.1186/s12959-026-00829-w)
Supplement: Supplementary file 1 — Supplementary Material 1 [file 12959_2026_829_MOESM1_ESM.docx]

**Supplementary Material**

Figure 1. The process of selecting AEs reports of antiangiogenic monoclonal antibodies from the standardized FAERS dataset

Table S1. Major algorithms used to assess potential associations

Table S2. List of preferred terms of thromboembolic events

Table S3. Signal strength of antiangiogenic monoclonal antibodies related TEEs compared with one another


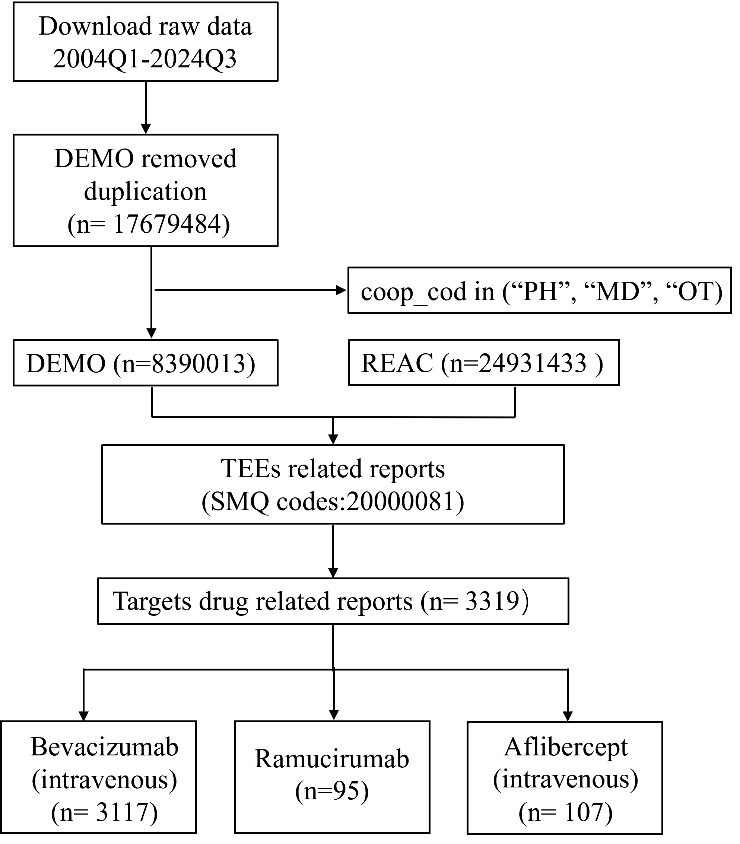


Figure 1. The process of selecting AEs reports of antiangiogenic monoclonal antibodies from the standardized FAERS dataset

Table S1. Major algorithms used to assess potential associations

| Algorithms | Equation | Criteria |
| --- | --- | --- |
| ROR | ROR = $\frac{a/b}{c/d}$ | ROR_025_>1 and a≥3 |
|  | ROR(95%CI)*=*$e^{ln(ROR\pm1.96\left( \sqrt{\frac{1}{a}+\frac{1}{b}+\frac{1}{c}+\frac{1}{d}} \right)}$ |  |
| IC | IC = log_2_($\frac{a/(a+b)}{(a+c)/N}$) | IC_025_>0 |
|  | IC(95%CI) = E(IC) ± 2$\sqrt{Var(IC)}$  E (IC) =log_2_$\frac{(a+1){(N+1)}^{2}}{(N+\gamma)(a+b+1)(a+c+1)}$  Var(IC)=($\frac{1}{log2}$)^2^[$\frac{N-a+\gamma-1}{\left( a+1 \right)(N+\gamma+1)}+\frac{N-(a+b)+1}{(a+b+1)(N+3)}+\frac{N-\left( a+c \right)+1}{(a+c+1)(N+3)}$]  $\gamma=\frac{{(N+2)}^{2}}{(a+b+1)(a+c+1)}$ |  |

Abbreviations: N, the number of reports; N=a+b+c+d; 95% CI, 95% confidence interval; ROR, reporting odds ratio; ROR_025_, the lower limit of the 95% CI of the ROR; IC, information component; IC_025_, the lower limit of the 95% CI of the IC; E (IC), the IC expectations; Var (IC), the variance of IC.

Table S2 List of preferred terms of thromboembolic events

| PE | "post procedural pulmonary embolism","pulmonary embolism", "pulmonary infarction","pulmonary microemboli","pulmonary thrombosis","pulmonary vein occlusion","pulmonary venous thrombosis","pulmonary veno-occlusive disease" |
| --- | --- |
| MI | "acute myocardial infarction","coronary artery embolism","coronary artery occlusion","coronary artery thrombosis","myocardial infarction","myocardial necrosis","papillary muscle infarction" |
| Cerebral ATE | "amaurosis","amaurosis fugax","basal ganglia infarction","basilar artery occlusion","basilar artery thrombosis","blindness transient","capsular warning syndrome","cerebellar artery occlusion","cerebellar artery thrombosis","cerebral artery embolism","cerebral artery occlusion","cerebral artery thrombosis","cerebral hypoperfusion","cerebrovascular stenosis","ischaemic cerebral infarction","ischaemic stroke","lacunar infarction","retinal artery embolism","retinal artery occlusion","retinal artery thrombosis","transient ischaemic attack" |
| VTE | SMQ=20000084 |
| ATE | SMQ=20000082 |
| Unspecified and mixed | SMQ=20000083 |
| Overall TEEs | SMQ=20000081 |

Table S3. Signal strength of antiangiogenic monoclonal antibodies related TEEs compared with one another

|  |  | ROR (95% CI) for BEV vs RAM | ROR (95% CI) for BEV vs ABL | ROR (95% CI) for ABL vs RAM |
| --- | --- | --- | --- | --- |
| ALL |  | 1.003(0.81-1.23) | 0.77(0.63-0.94) | 0.77(0.58-1.02) |
| PE |  | 0.95(0.58-1.56) | 1.02(0.59-1.77) | 1.07(0.52-2.24) |
| MI |  | 1.64(0.61-4.4) | 0.44(0.25-0.77) | 0.27(0.09-0.82) |
| Cerebral ATE |  | 0.57(0.3-1.08) | 0.83(0.37-1.87) | 1.46(0.53-4.01) |
| VTE |  | 1.11(0.78-1.58) | 1.15(0.78-1.68) | 1.03(0.62-1.73) |
| ATE |  | 0.77(0.55-1.09) | 0.6(0.43-0.83) | 0.78(0.49-1.24) |
| Unspecified and mixed |  | 1.13(0.8-1.58) | 0.68(0.51-0.91) | 0.61(0.39-0.94) |

BEV, bevacizumab; RAM, ramucirumab; ABL, aflibercept.
